# The Influence of Structure of Multilayer Woven Fabrics on Their Mechanical Properties

**DOI:** 10.3390/ma14051315

**Published:** 2021-03-09

**Authors:** Ewa Witczak, Izabela Jasińska, Iwona Krawczyńska

**Affiliations:** Lukasiewicz Research Network Textile Research Institute, 92-103 Łódź, Poland; izabela.jasinska@iw.lukasiewicz.gov.pl (I.J.); iwona.krawczynska@iw.lukasiewicz.gov.pl (I.K.)

**Keywords:** multilayer woven, conveyor belts, bending rigidity, woven structure

## Abstract

Multilayer woven fabrics used for conveyor belts must be characterized by high mechanical strength. The design process of multilayer woven fabrics for such application requires taking into account the structural characteristics of the fabric, which allows to adjust the final product properties to the dedicated use. The geometry of warp threads—means stuffer and binding is the decisive aspect, which influences the strength properties of multilayer woven fabrics and materials made with their use as well. The aim of this work was to examine the possibility of shaping mechanical strength and bending rigidity of multilayer woven fabrics by changing the order of introducing weft threads into individual layers. The eight variants of multilayer woven fabrics were manufactured using laboratory harness loom. They were produced using different structural models in two weft variants, then tested. The mechanical features were determined, such as breaking force, recovered and unrecovered elongations in cyclic tensile test, stiffness rigidity. The analysis of the obtained results confirmed, that both the model and the order in which the weft threads were introduced into individual layers influence the mechanical strength and bending rigidity of multilayer woven. It was found, that the strength properties characterized by the above mentioned indicators are influenced by the number of threads weaved as both the stuffer and binding warp.

## 1. Introduction

Textiles are a group of products which, thanks to their properties and diverse structures, are used in many areas, including technical ones. The usage of textiles as structural elements, thanks to their ability to shape their structure and properties, allows to provide engineering constructions meeting of high strength requirements. The one of the group of textile materials, that are used in technical applications, are multilayer woven fabrics (MLW). The MLW are structures consist of a great number of layers connected to each other by an additional warp or arrangement of warp threads. Multilayer woven fabrics are most often used for drive belts, conveyor belts, structural composite reinforcements and others. Conveyor belts are usually used in coal mines, cement and lime industries, paper and sugar factories, agriculture, and power plants [1,2,3].

The right choice of the belt is crucial for the stability of the whole installation. The belt, regardless of its type and purpose, has solid elements, which consist of: spacers made of fabric impregnated with latex solution, carrying pulling force, and rubber covers, protecting the core against damage, affecting the life of the tape [4,5]. All materials used in the production of conveyor belts are characterized by a strong non-linear mechanical behaviour. During an operation on the conveyor, the belt is subjected to loads that change over time [6].

The safety coefficient of the conveyor belt system changes with the type and size of the material to be transported, the methods of material loading, the belt construction and installation of the conveyor. It is assumed that the working load is about 10–15% of the breaking force of the conveyor belt. In order to avoid slippage between the belt and the drive drum, meeting the criterion of low elongation and high dimensional stability in the longitudinal direction of the belt is crucial. The ability of the conveyor belt to form a gutter in the transverse direction is also important. The number of spacers and the distance between them, as well as the woven structure and its bending rigidity influence the belt ability to form a gutter [7]. When determining the character of the belt, the bending rigidity and how the belts fits to the entire conveyor system determines the character of belt [8,9,10,11].

A special conveyor belt consists of three zones, symmetrically distributed along the longitudinal belt direction, where edge zones rigidity increase or decrease in relation to the middle part [4,5].

The bending behaviour of reinforcement is a multiscale hierarchical problem. In many cases, especially during advance composites analysis numerical method was used to assess stiffness, bending rigidity and mechanical strength of designed products. For example, the decomposition of various types thin-walled members was investigated using modal finite strip method [12]. Despite of usually high tensile strength of composites, joints could be theirs the weakest part. The analysis of mechanical behaviours both proper and defected T-joints locked inside the composited structures was presented in research work [13]. Moreover, the assessment of mechanical properties located inside composites elaborated woven structures could be carry out by manufacturing and testing them. The fibrous structures, consisting from many thin, single fibres, are hard to investigate numerically. At the microscopic scale, yarn is made up of fibres that interact with each other, but the bending rigidity of the yarn is not the sum of the bending rigidity of the fibres. The relationship between the mechanical behaviour of fabric, yarns, and fibres is complex [14,15,16,17]. Bending rigidity in particular determines the correct shape of wrinkles that appear during draping at macroscale [18] or macroscopic extension of fabrics. In comparison to continuous materials, bending and tension rigidities of fibrous materials are not directly related. Consequently, extra experiments are usually required to determine bending rigidity of the material [19,20,21,22,23,24,25]. The high rigidity of the fabric influences enlarging the rigidity of the conveyor belt, but their transverse flexibility plummeted. The stresses generated in the conveyor structure at the moment of the return of the belt could be minimalized by choosing the optimal woven structure, e.g., with gaining high transverse flexibility, as well as by eliminating multiple spacers [26,27].

In a single woven, the bending modulus is slightly dependent on the direction of the tested samples. It has been shown that the higher the number of covers in a given direction, the lower the bending rigidity in that direction. Incrementally, the number of crossings in the fabric and stiffening the liners evenly increases the rigidity of the woven [28]. The basic parameters of the internal structure of fabrics change significantly under the influence of heat treatment during vulcanization and formation of conveyor belts. Moreover, the weave of the fabric significantly influences its internal structure and indirectly the susceptibility to higher temperature. These changes probably have a significant influence on mechanical properties of the woven at every stage of conveyor belt production. In the case of high strength conveyor belts used in difficult working conditions, intensive research is conducted to identify new types of fibres, weave, and spacing systems, that can meet the growing demands of end users [29].

The geometry of the warp threads, both stuffer and binding ones, is a decisive aspect, which affects the mechanical strength of MLW with identical arrangement of weft threads and materials made of them, including composites. 

The main goal for carried out works was to investigate the possibility of shaping the mechanical strength properties and bending rigidity of MLW by sequence insertion of the warp threads into individual layers. In previous research works, the way the MLW layers are bonded by means of the warp binding was analysed [30]. It was found that warp introducing order was responsible for load transfer characteristic and can influence tensile strength of products significantly. 

The continuous development and new applications of such products and growing expectations of its end users cause the continuous development of MLW structures and dedicated them measurement methods [31].

## 2. Materials and Specimen Preparation

The four basic structural models of MLW are known, based on the geometry of binding threads. The course of the binding threads, i.e., the method of binding significantly influences the internal structure of the woven fabric. The distribution of the binding threads also affects the quantity and packing of the two remaining basic systems. The structures of multilayer woven were classified into four models taking into account the geometry of binding warp thread’s course. 

**Figure 1:**
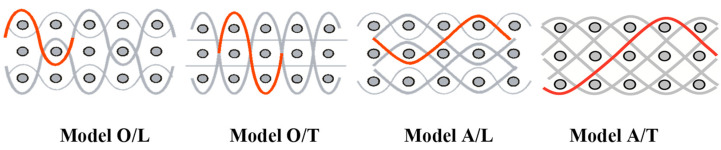
Structural models of multilayer woven

Structural models of multilayer woven [32] (Figure 1):

− model O/L—orthogonal interlock/layer to layer binding,
− model O/T—orthogonal interlock/through-thickness binding,
− model A/L—angle interlock/layer to layer binding,
− model A/T—angle interlock/through-thickness binding.

**Figure 2:**
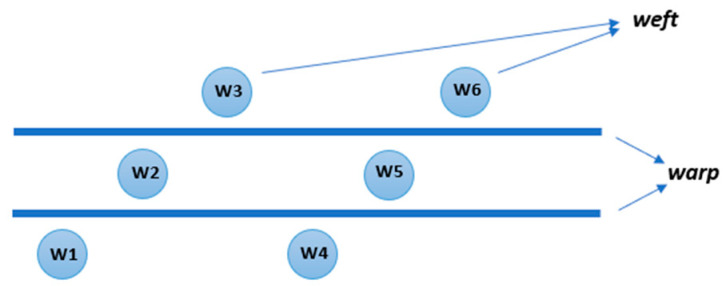
Subsequent model of wert insertion.

**Figure 3:**
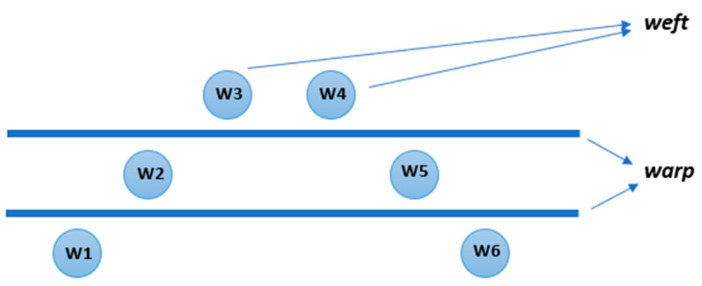
Shifted model of wert insertion.

The order of wefts to be inserted:

k—subsequent—from the bottom to the top layer, presented in Figure 2;

p—shifted—from lower to upper layer—from upper to lower layer, shown in Figure 3.

For each structural model, two versions of multilayer woven, differ in case of thread insertion order into the individual layers have been developed (Table 1).

The MLW fabrics were manufactured of 1060 dtex with 256 filaments, high strength continuous filament polyester yarn with reduced elongation. The yarn twist was 60 twists/m. The tensile properties of yarn were measured, results in Table 2 was shown.

**Figure 4:**
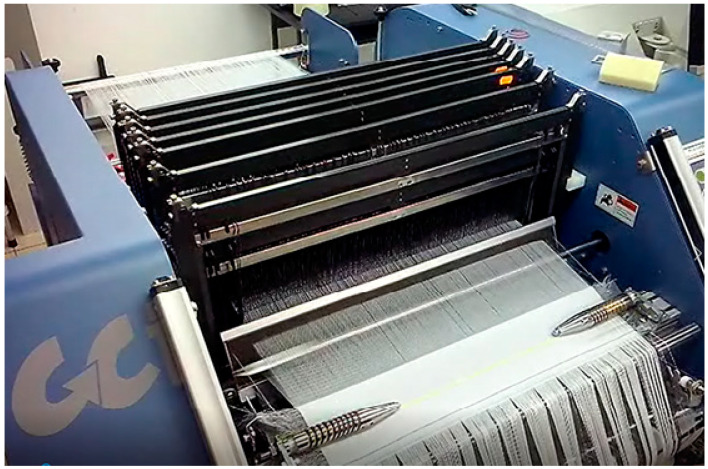
The laboratory harness loom during manufacturing of MLW.

The multilayer woven were produced on SW550 type laboratory weaving station, consisting of an automatic warping machine and SL8900S harness loom (Figure 4), equipped with two warp shafts while the following constant technological parameters remain constant:
number of layers5number of warp threads2400width of weaving40 cmnumber of warp threads60 threads/1 cmnumber of weft threads25 threads/1 cmwarp tension (value set on the loom)15 kg

## 3. Mechanical Tests 

The basic structural parameters of produced MLW fabrics were tested. The mass per unit area according to PN-EN 12127:2000 [33] was determined thickness according to PN-EN 5084:1999. The crimp factor of stuffer and binding warp threads [34] following the Equation (1) was calculated:

(1) $$w_{o}=\frac{lrz_{o}-lp_{o}}{lp_{o}}100$$

where:

$w_{o}$—crimp factor, %;
$l_{rz_{o}}$—the actual length of the warp thread taken from woven section, mm;
$l_{p_{o}}$—the section length of woven fabric within the warp thread is present, mm.

During the tensile tests of manufactured fabrics, the maximum force F_max_ and elongation at this force were determined in accordance with PN-EN ISO 13934-1:2013-07 [35]. 

For further tests purpose it was assumed, that during use, the forces acting on the fabric structure constitute about 70% of the F_max_. The fabrics were subjected to five cycles of stress and relaxation up to 70% F_max_. The values of recovered and un-recovered elongations were determined in the 4th test cycle. Additionally, the values of elongations at the force of 35% F_max_ were determined in three consecutive measuring cycles (fatigue test). 

The samples were conditioned and tested in the atmosphere: the temperature of 20 ± 2 °C and relative humidity 65 ± 5%.

The bending rigidity of fabrics was performed using the fixed angle method according to PN-73/P-04631. The test method is based on the relationship developed by Peirce, between the length of woven sample from the fixed point to leading edge deformed under its own weight and bending rigidity of woven fabric.

### 3.1. Test Results

#### 3.1.1. Structural Parameters

The results obtained during mentioned test are presented in Table 3.

In fabric I, in which the threads are introduced successively into the layers, the mass per unit area is almost 9% greater than in fabric Ia, in which the threads are introduced in an shifted order. It is related to the increase in the warp thread crimp factor. In case of fabrics: II, III, and IV, the area weights are lower in the variant of the subsequent introduction of threads compared to fabrics in which the threads are introduced in an shifted order, respectively, for IIa by 1%, IIIa by 2.9%, and IVa- by 2.8%.

The highest thickness in both weft variants, reaching 2.35 and 2.43 mm, respectively, for fabric III was achieved. Mentioned fabrics are characterized by the lowest values of the warp thread crimp factor (3–5 and 2–4%, respectively) and the lowest values of mass per unit area. The smallest thickness for fabric II was obtained, in both variants (II and IIa), achieving 1.72 and 1.70 mm, respectively. The crimp factor of stuffer warp threads and binding warps differs significantly from 3 to 19% (fabric II) and from 4 to 20% for fabric IIa, respectively. The crimp factor of weft threads is greater in the case of shifted weft variant, for all fabrics. The considerable differences occur in fabrics produced according to the O/T and O/L models, but in case of A/T and A/L fabrics models they are less significant.

#### 3.1.2. Mechanical Properties of the Woven Fabrics

**Figure 5:**
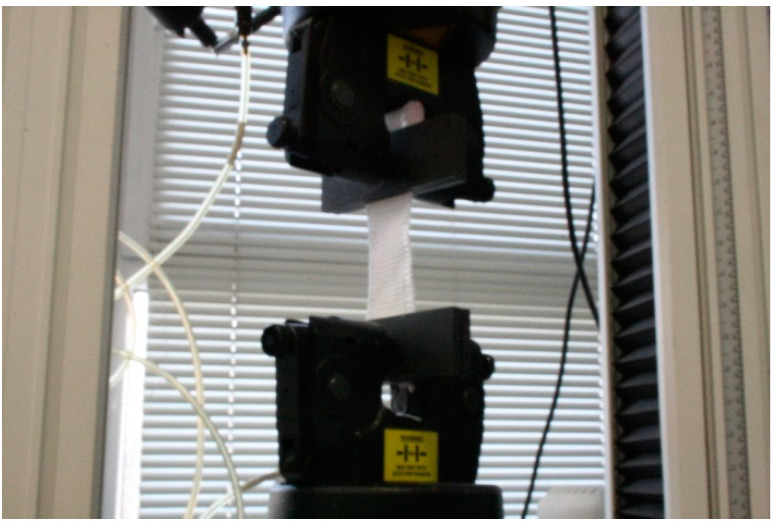
MLW sample during tensile test.

The test results obtained for mechanical properties of woven fabrics are presented in Table 4. The measurements were carried out using Instron 3367 tensile machine (Figure 5) equipped with tensile sensors of following capacity: 10 and 30 kN.

The both variants of fabric I (I and Ia) showed the lowest values of maximum force F_max_, reaching 4982 and 4862 N, respectively. The highest values of F_max_ were achieved by fabrics II and IIa (10,500 N). The reason, that F_max_ values, recorded for fabric I and IIa were the lowest ones was the way of both warps inside A/T model. The stuffer and binding warps were fixed not enough firmly in woven structure, creating the long paths between the thread’s jams in fabric. Therefore, the load was transmitted in the simply layout, warp threads were elongated along relatively long distances in fabric structure, being more fragile to damage. Typically, inside this structure only the part of warps works simultaneously, so if they broke, the fabrics failed. These woven fabrics were made according to the models in which the layers binding took place through the whole thickness of the fabric (i.e., T). On the other hand higher values of maximum force occur for the shifted warp variant in the case of fabrics performed in accordance with the L binding layer model, means III/IIIa and IV/IVa woven fabrics. 

The binding model together with warp order variant shown an opposite effect on elongation value at the force F_max_. In case of test result obtained for A model fabrics with angled binding, higher elongation values occur when subsequent warp order is used, compared to shifted one.

The fabrics III and IV in both warp order variants present the best elastic properties. The recovered elongation determined for fabrics III and IV reach up to 72%, compared to 58 and 61% obtained for fabric I. The warp order variant does not influence recovered elongation significantly. In structures designed for conveyor belts, which operates in continuous mode and under heavy load, it is important to evaluate the elongation in the fatigue test. The values of elongation at 35% F_max_ in the 3rd tensile cycle marked ɛ_35,_ correspond to the fatigue characteristics of the fabric. The highest values of ɛ_35_ was 24%, reached by fabric I and 21%—reached by fabric Ia. The lowest ɛ_35_ values 11 and 12% were achieved by fabrics III and IIIa, respectively. The lowest increase in elongation values from first to third cycle shown fabrics I and Ia, 26 and 30%, respectively, while for the other fabrics this enlargement is higher, especially for subsequent weft order, rising to even 48% for fabric II.

**Figure 6:**
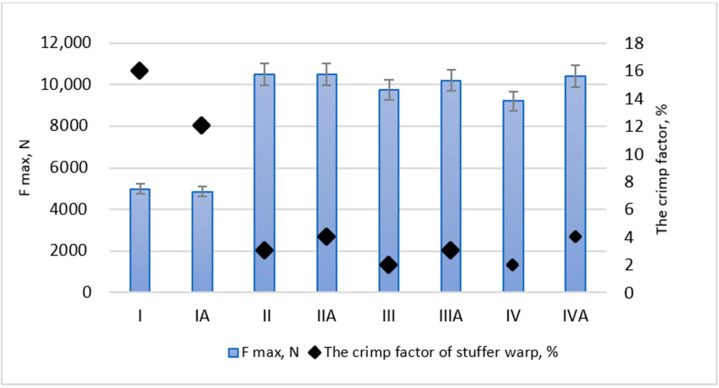
The relationship between Fmax and the crimp factor of stuffer warp.

**Figure 7:**
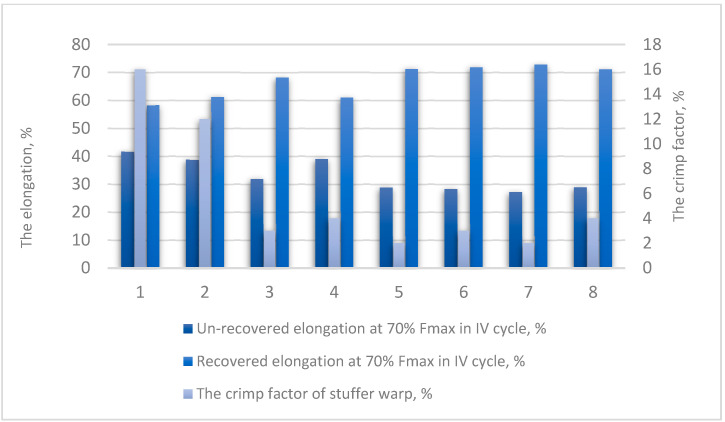
Summary of elongation and the crimp factor for tested fabrics.

It was found, that the insertion way of stuffer warp threads influences strength characteristics the most. Taking into account the relationship between the F_max_ and the stuffer thread crimp factor (Figure 6), a firm negative correlation was found (correlation coefficient *r* = −0.93). The value of elongation at F_max_ and the crimp factor of stuffer warp is characterized by a strong positive correlation (correlation coefficient *r* = 0.87). The values of recovered elongation at 70% F_max_ in the 4th cycle and the crimp factor of stuffer warp (Figure 7) are also correlated (correlation coefficient *r* = −0.82).

#### 3.1.3. Binding Rigidity

For MLW characterized by the lowest and highest values of elongation at 35% F_max_, i.e., fabrics I, Ia, III, and IIIa the bending rigidity was determined. In order to analyse the influence of the structural model, the bending rigidity was additionally determined for fabric II (Table 5).

For fabrics dedicated for conveyor belts, the bending rigidity along the weft is an important parameter. The bending rigidity determines the structure ability to take the shape from a conveyor. The highest values of the rigidity of the tested fabrics along the weft were found in case of fabric I and Ia. Additionally, the difference between the bending rigidity along weft and warp directions in these fabrics was the greatest. The fabric III in both warp order variants has the lowest values of bending rigidity along the weft. Moreover, in this case, bending rigidity in both warp and weft directions are very similar. At the same time, there was no effect of warp order on fabric rigidity for fabric III. The compared fabrics I and III are manufactured according to model A (angle interlock) and significant differences in bending rigidity come from the method of binding (L or T). Binding type L (layer to layer) caused a decrease in bending rigidity along the weft.

Comparing the values of the analysed parameter determined for fabrics I and II (models A and O), it can be concluded that for model A (angle interlock) the bending rigidity along the warp is much lower, despite along the weft being at the same level.

**Figure 8:**
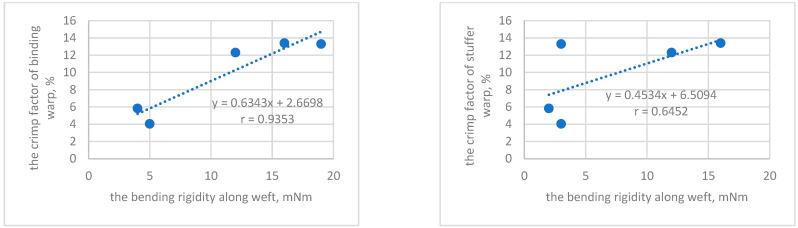
The relationship between bending rigidity along weft and the crimp factor of warps.

The significant influence the crimp factor of binding warp on bending rigidity was found, the correlation coefficient reached *r* = 0.94 (Figure 8). Moreover, the correlation between bending rigidity along weft direction and the crimp factor of stuffer warp was determined (*r* = 0.64). 

## 4. Conclusions

The multilayer woven fabrics were made according to different structural models in two warp order variants and was tested for of determination their structural parameters, tensile strength properties and bending rigidity. The analysis of the obtained results confirmed that both the model and the order in which the warps were introduced into individual layers affect the mechanical properties and bending rigidity of MLW. In terms of the weft bending rigidity values along recovered elongation and breaking force, the best results were obtained for fabrics III and IIIa. A slight improvement in the weft bending rigidity, the value of recovered elongation at 70% F_max_ in the 4th cycle, as well as the maximum force (F_max_) by using shift weft order was obtained. It was found that the crimp factor values determined for both warp threads (stuffer and binding) have an influence on tensile properties of MLW, characterized by F_max_, recovered elongation and bending rigidity. In the individual models and variants of weft order, the crimp of warp threads varies and is related to the geometry of threads course. The evaluation of test results shown, that mechanical strength and bending rigidity of MLW could be shaped by their construction, especially by way in which the warps were interlaced. The design process of multilayer woven fabrics for technical applications requires understanding and evaluating the structural characteristics of the fabric, which allows the product properties to be adapted to the dedicated application. The results of carried out works were an input data for further search for optimal MLW properties. They include conveyor belts core structures with different areas desirable stiffness.

## Figures and Tables

**Table 1:** The modified weaves of MLW (multilayer woven fabrics) and their cross sections along the warp.

Sample Description	Weave Scheme	Weave Visualization	Woven Fabric Picture
I model A/T/k Ro—6, Rw −18	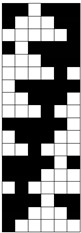	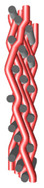	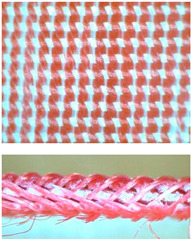
Ia model A/T/p Ro—6, Rw −18	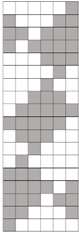	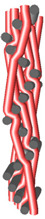	
II model O/T/k Ro—10, Rw −10	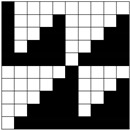	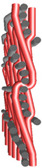	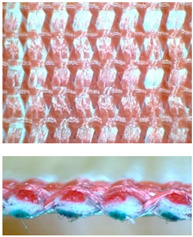
IIa model O/T/p Ro—10, Rw −10	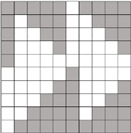	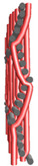	
III model A/L/k Ro—10, Rw −20	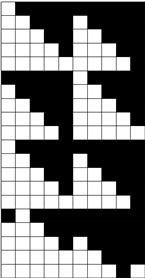	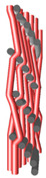	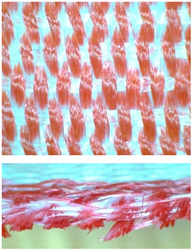
IIIa model A/L/p Ro—10, Rw −20	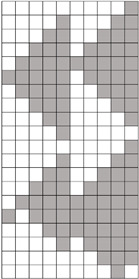	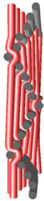	
IV model O/L/k Ro—6, Rw −10	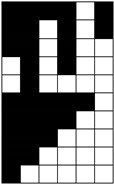	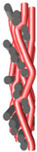	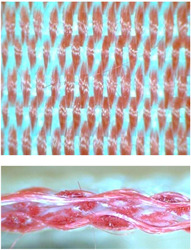
IVa model O/L/p Ro—6, Rw −10	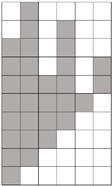	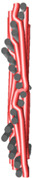	

**Table 2:** The tensile test results for used yarn.

Parameter	Standard	Mean Value	Coefficient of Variation
Breaking force F_B_, cN	PN-EN ISO 2062:2010	6601 ± 184	2.79
Elongation at break, %	PN-EN ISO 2062:2010	13.3 ± 0.9	6.55
Elongation after I cycle, mm:	PN-P-04667:1984	4.1	0.89
- recovered		3.5	0.83
- un-recovered		0.6	9.76
Elongation after V cycles, mm:	PN-P-04667:1984	4.64	0.78
- recovered		3.5	1.27
- un-recovered		1.2	9.76

**Table 3:** Summary of structural parameters of fabrics.

Sample		Structural Parameters of Fabrics					
		Mass Per Unit Area, g/m^2^	Thickness, mm		Crimp Factor of Warp Threads, %		Crimp Factor of Weft Threads, %
					Stuffer	Binding	
I	A/T/k	1058	1.91	±0.03	16	16	0.77
Ia	A/T/p	973	1.95	±0.12	12	12	0.97
II	O/T/k	967	1.72	±0.06	3	19	1.50
IIa	O/T/p	977	1.7	±0.03	4	20	2.13
III	A/L/k	923	2.35	±0.03	2	4	1.84
IIIa	A/L/p	950	2.43	±0.04	3	5	1.87
IV	O/L/k	967	1.85	±0.03	2	4	0.80
IVa	O/L/p	995	1.86	±0.08	4	6	3.84

**Table 4:** Summary of test results of mechanical properties of the woven.

Parameter	Sample							
	I	Ia	II	IIa	III	IIIa	IV	IVa
	A/T/k	A/T/p	O/T/k	O/T/p	A/L/k	A/L/p	O/L/k	O/L/p
Fmax, N	4982	4862	10,500	10,500	9756	10,196	9223	10,414
Elongation at Fmax, %	17.80	14.40	11.20	12.60	13.80	13.00	12.20	12.90
Elongation at 35% Fmax, mm in I cycle	19.0 ± 0.8	16.0 ± 0.6	8.1 ± 0.1	12.0 ± 0.4	7.9 ± 0.2	8.9 ± 0.1	9.0 ± 0.0	9.9 ± 0.2
in II cycle	23.0 ± 1.1	20.0 ± 0.8	11.0 ± 0.2	15.0 ± 0.5	10.0 ± 0.3	11.0 ± 0.2	12.0 ± 0.2	13.0 ± 0.3
in III cycle	24.0 ± 1.2	21.0 ± 0.7	12.0 ± 0.2	15.0 ± 0.5	11.0 ± 0.4	12.0 ± 0.3	12.7 ± 0.2	13.3 ± 0.3
Un-recovered elongation at 70% Fmax in IV cycle, %	41.7 ± 1.3	38.8 ± 0.3	31.8 ± 1.0	39.0 ± 1.0	28.8 ± 0.4	28.2 ± 0.5	27.2 ± 0.3	28.9 ± 0.5
Recovered elongation at 70% Fmax in IV cycle, %	58.30	61.20	68.20	61.00	71.20	71.80	72.80	71.10

**Table 5:** Results of bending rigidity test for MLW fabrics.

Sample		Bending Rigidity along the Warp, mNm	Bending Rigidity along the Weft, mNm
I	A/T/k	1.91	13.4
Ia	A/T/p	1.76	12.3
II	O/T/k	9.10	13.3
III	A/L/k	4.04	5.83
IIIa	A/L/p	5.51	4.05

## Data Availability

The data presented in this study are available on request from the corresponding author. The data are not publicly available due to its paper form (test reports, detailed data coming from tensile machine).
